# Expression of RAGE and HMGB1 in Thymic Epithelial Tumors, Thymic Hyperplasia and Regular Thymic Morphology

**DOI:** 10.1371/journal.pone.0094118

**Published:** 2014-04-04

**Authors:** Bernhard Moser, Stefan Janik, Ana-Iris Schiefer, Leonhard Müllauer, Christine Bekos, Anke Scharrer, Michael Mildner, Ferenc Rényi-Vámos, Walter Klepetko, Hendrik Jan Ankersmit

**Affiliations:** 1 Department of Thoracic Surgery, Division of Surgery, Medical University Vienna, Vienna, Austria; 2 Department of Pathology, Medical University Vienna, Vienna, Austria; 3 Christian Doppler Laboratory for the Diagnosis and Regeneration of Cardiac and Thoracic Diseases, Medical University Vienna, Vienna, Austria; 4 Department of Dermatology, Medical University Vienna, Vienna, Austria; 5 Department of General and Thoracic Surgery, National Institute of Oncology, Budapest, Hungary; University of Miami, United States of America

## Abstract

Recently, a role of the receptor for advanced glycation endproducts (RAGE) in myasthenia gravis was described. RAGE and its ligand high mobility group box 1 (HMGB1) play key roles in autoimmunity and cancer. To test whether these molecules are involved in patients with thymic abnormalities we applied immunohistochemical analysis in 33 cases of thymic epithelial tumors, comprising 27 thymomas and 6 thymic carcinomas, and 21 nonneoplastic thymuses. Both molecules were detected in neoplastic epithelial cells: RAGE staining was most intense in WHO type B2 thymomas and thymic carcinomas (p<0.001). HMGB1 nuclear staining was strongest in A and AB, and gradually less in B1 = B2>B3>thymic carcinoma (p<0.001). Conversely, HMGB1 cytoplasmic staining intensities were as follows: A and AB (none), B1 (strong), B2 (moderate), B3 and thymic carcinoma (weak); (p<0.001). Fetal thymic tissue showed a distinct expression of RAGE and HMGB1 in subcapsular cortical epithelial cells which was found in 50% of myasthenic patients. Furthermore RAGE and HMGB1 were expressed in thymocytes, macrophages, Hassall's corpuscles, thymic medulla, and germinal center cells in myasthenic patients. Immunohistochemistry results were complemented by systemic measurements (immunosorbent assay): serum levels of soluble RAGE were significantly reduced in patients with epithelial tumors (p = 0.008); and in invasive tumors (p = 0.008). Whereas RAGE was equally reduced in thymic hyperplasia and epithelial tumors (p = 0.003), HMGB1 was only elevated in malignancies (p = 0.036). Results were most pronounced in thymic carcinomas. Thus, RAGE and HMGB1 are involved in the (patho-)physiology of thymus, as evidenced by differentiated thymic and systemic expression patterns that may act as diagnostic or therapeutic targets in autoimmune disease and cancer.

## Introduction

Thymomas and thymic carcinomas are rare malignant neoplasms of thymic epithelial origin (thymic epithelial tumors, TETs) with an overall incidence of 0.15 per 100,000 person-years [1]. They are the most frequent anterior mediastinal tumors in adults. The 2004 update of the World Health Organization (WHO) histological classification of TETs distinguishes 6 main types based on the morphology of the neoplastic epithelial cells and the amount of intratumoral nonneoplastic lymphocytes: thymoma types A, AB, B1, B2, B3 and thymic carcinomas (TC) [2]. The invasiveness of TETs is widely classified with the Koga modification of the pathological Masaoka staging system [3]. The Masaoka-Koga staging system has been shown to have prognostic significance by many authors and also in our patient cohort [4]. There are neither established risk factors [5] nor biomarkers for screening of TETs that could help clinicians distinguish TETs from benign enlargement of the thymus, namely thymic hyperplasia (TH). TH was defined as a non-neoplastic thymic change with an increase in constituent cells [6]. Two types can be distinguished. True thymic hyperplasia (TTH) is characterized by increased weight and size of the thymus with regular microscopic histologic architecture, whereas follicular (or lymphoid) thymic hyperplasia (FTH) is defined by the presence of lymphoid follicles with germinal centers in the thymic medulla [7].

Myasthenia gravis (MG) is a rare neurological autoimmune disorder characterized by autoantibodies against the acetylcholine receptor (AChR) or other proteins of the neuromuscular junction. The functional loss of AChR ultimately leads to impaired neuromuscular transmission and results in characteristic fluctuating muscle weakness [8], [9]. Immunosuppressive therapy is associated with better survival and quality of life. About 80% of patients with MG show thymic abnormalities, including TTH, FTH or TETs. Surgical thymectomy has curative intent and demonstrates clinically relevant symptom improvement or even remission of MG in a high proportion of patients [10]. There is a unique association of TETs with paraneoplastic syndromes and autoimmune disorders, such as hypogammaglobulinemia, aplastic anemia, and most frequently MG [11]. TETs can be found in approximately 15% of patients with MG, while approximately 35% of patients with TETs have MG [12].

The receptor for advanced glycation endproducts (RAGE), a member of the immunoglobulin superfamily, encoded in the major histocompatibility complex class III region, is composed of a variable domain, two constant domains, a transmembrane domain and a cytoplasmic tail [13]. RAGE is an activating signal transduction receptor interacting with multiple ligands, such as proinflammatory advanced glycation endproducts (AGEs), the products of nonenzymatic glycation and oxidation of proteins [14]; S100/calgranulins [15], [16]; and high-mobility group box 1 (HMGB1) [17]; to amplify inflammatory responses. RAGE is expressed by cells involved in immune responses: monocytes/macrophages [18], CD4^+^ and CD8^+^ T cells [19] and dendritic cells [20]. On T cells, the receptor is inducibly upregulated and required for efficient antigen specific priming. RAGE engagement has a critical role for cognate dendritic cell–T cell interactions [21]. Furthermore, RAGE contributes to allogeneic T cell proliferation and the rejection of solid organ allografts [22]. Soluble (s) RAGE (sRAGE) is the extracellular domain of RAGE that acts as a decoy for the above described ligands. There is an alternative splice variant of the RAGE gene, called endogenous secretory RAGE (esRAGE) that is actively secreted, whereas sRAGE results from proteolytic cleavage of the cell surface bound full-length RAGE receptor [23]. The RAGE ligand HMGB1 is a non-histone chromosomal protein which functions as a DNA chaperone. Outside the nucleus, it has diverse functions in the cytosol, mitochondria, at the cell surface and the extracellular fluid. The molecule is composed of two homologous DNA binding domains and an acidic tail. Different binding domains for its receptors: RAGE, Toll-like receptor 4 (TLR4) and a p53 transactivation domain have been identified. Once HMGB1 is released from the cell it acts as a signaling molecule, namely a damage-associated molecular pattern molecule (DAMP) [24], [25].

The current knowledge about the pathophysiologic involvement of the RAGE axis in cancer has recently been reviewed elsewhere [24], [25]. Briefly, RAGE has been implicated in enhancing chronic inflammation laying the ground for growth of epithelial malignancies [26], mediating resistance of tumor cells to hypoxia [27], tumor invasion and metastasis [28]. HMGB1 has been ascribed a dual role in cancer development and therapy, promoting cell survival as well as death by interfering with multiple signaling pathways, e.g. in inflammation, proliferation, apoptosis, autophagy and metastasis [24]. Aberrant expression of RAGE or RAGE ligands in human patients with malignancies has been reported in e.g. pancreatic cancer [29], clear cell renal cell carcinoma [30], colorectal cancer [31], glioblastomas [32], primary nasopharyngeal carcinomas and their metastases to lymph nodes [33], cervical squamous cell carcinoma [34], gastric adenocarcinoma [35], and non-small cell lung cancer [36].

We have recently shown that both soluble receptors, sRAGE and esRAGE, are significantly reduced in serum of patients with MG without TETs [9]. Clinical categorization of patients with MG according to the distribution of involved muscles, disease onset or acetylcholine receptor antibody serum status did not reveal a clinical disease entity with physiological levels of soluble RAGE receptors. Moreover, patients receiving current immunosuppressive or symptomatic disease-specific pharmacological therapy did not show modulated RAGE levels compared to patients not receiving these treatments.

Dysregulation of immunity in patients with MG, the close association of thymic abnormalities (including TETs) and MG, and the involvement of RAGE and HMGB1 in different cancers and autoimmune diseases sparked our interest in TETs, TH and thymic physiology. The present study sought to investigate a role for the RAGE pathway in thymic development, benign TH and malignant tumors of the thymic epithelium in human patients.

## Materials and Methods

### Ethics Statement

Ethical approval was obtained from the Medical University Vienna review board on human research. Written informed consent was obtained from all patients and volunteers participating in this study

### Subjects

All adult patients included in this study underwent extended thymectomy. The indications for surgery were the following: (1) radiological suspicion of a mediastinal mass, (2) histologically verified thymoma/thymic carcinoma or (3) myasthenia gravis. All patients underwent resection at the department of thoracic surgery at the Medical University Vienna between 2009 and 2013. The immunohistochemical study of TETs was based on six cases of primary TCs, 6 cases of WHO type A thymomas, 3 cases of WHO type AB thymomas and 18 cases of WHO type B thymomas (6 B1, 6 B2 and 6 B3). No patient with TC had MG in this study. TCs were subtyped as squamous cell carcinomas. Fifty percent of all cases for WHO types A, B1, B2 and B3 were from patients with MG. Specimens of regularly involuted adult thymus were identified from patients undergoing minimally-invasive extended thymectomy for radiological suspicion of a mediastinal mass (neither of these patients had a malignancy nor TH). The diagnosis of true thymic hyperplasia or regular for age thymic tissue was based on size and weight of the resected thymus - none of these patients had a thymoma/thymic carcinoma. In patients with non-thymomatous MG (n = 11) four patients received immunosuppression (three patients: corticosteroids, one patient: methotrexate). Seven patients received symptomatic treatment with pyridostigmine. Fetal thymic tissue for histological processing was obtained at autopsy. There were two artificial abortions and two spontaneous intrauterine deaths. The shortest and the longest time from death until autopsy for fetal thymic specimens were 41 and 96 hours. There were no signs of autolysis of fetal thymic tissue. There was no difference in staining pattern or intensity between the specimens. Histological diagnoses and classification of TETs and thymic tissues in this study were routinely performed at the institutional department of pathology at the medical university of Vienna. Serum samples of patients were collected one day before surgery and stored at −80°C until analysis.

### Immunohistochemistry

Formaldehyde-fixed and paraffin embedded human thymic or thymoma/thymic carcinoma tissue was retrieved from the institutional department of pathology. Sections, 2 μm in thickness, were prepared according to routine protocols. Briefly, sections were baked for 1 hour at 55°C, deparaffinized in three xylenes and rehydrated in ethanol as follows: 2× 100%, 1× 95%, 1× 90%, and 1× 70%, followed by PBS. Antigen retrieval was performed by boiling slides at 600 watt (3× 5 min) in a microwave oven using citrate buffer at pH 6.0 (Target Retrieval Solution, Dako, USA). Endogenous peroxidase activity was blocked by applying hydrogen peroxide 0.3%. Sections were incubated with 2% bovine serum albumin or blocking serum of the same species as the biotinylated secondary antibody to deplete unspecific protein-protein interactions. Sections were stained using affinity-purified polyclonal goat anti-human RAGE IgG (R&D Systems, Minneapolis, MN, USA) or monoclonal mouse anti-human HMGB1 IgG_2b_ (R&D Systems) and biotinylated anti-goat IgG or anti-mouse IgG secondary antibodies (Vector Laboratories, Burlingame, CA, USA). Immunoreactivity was amplified using biotin-avidin-peroxidase conjugates (Vectastain ABC kit, Vector Laboratories). 3,3′-diaminobenzidine was used as chromogen (DAB Peroxidase substrate kit, Vector Laboratories). Counterstaining was performed using Mayer's hematoxylin. Slides were dehydrated with ethanol: 1× 95% for 1 min, 1× 100% for 6 min and cleared in n-Butanol before mounting (Pertex Mounting Media, Leica Microsystems, Germany).

Immunohistochemistry for molecules markers of epithelial origin, keratins 5 and 14 were used on sections of fetal thymic tissue using the automated Ventana Benchmark® platform (Ventana Medical Systems, Tucson, AZ, USA) according to routine protocols of the institutional department of pathology. Sections were stained with monoclonal mouse anti-human cytokeratin 5 and 14 IgG (LH8, Abcam, CA, USA). Heat pre-treatment was conducted in Ultra cell conditioner number 1 buffer (Ultra CC1; pH 6). Color was developed with Ultraview Universal Detection DAB-kit (Ventana Medical Systems). Immunohistochemical staining for RAGE and HMGB1 was reproduced with the described automated system.

Omission of primary antibody served as negative control. Analysis and image documentation was done with Axio Imager 2 microscope and AxioVision software (Carl Zeiss International, Germany).

### Evaluation of immunoreactivity

Analysis of immunoreactivity was performed by two observers blinded to the diagnosis of MG and antibodies used for staining. A published scoring system was adopted [34]. We assigned a score from 0 to 3 to assess staining intensity for RAGE or HMGB1 cytoplasmic or nuclear expression in TETs (0, no staining; 1, weak; 2, moderate; 3, strong). For the analysis of staining intensity of areas or cells in nonneoplastic thymic tissue we used an abbreviated version (“ ”, no staining; “+”, positive; “++”, strongly positive). The percentage of epithelial tumor cells displaying staining for cytoplasmic or nuclear RAGE or HMGB1 was determined and calculated as average of six high power fields per specimen.

### Detection of serum proteins

To test the hypothesis that RAGE and HMGB1 are involved systemically in patients with thymic disease processes, we employed enzyme-linked immunosorbent assays (ELISA) for the detection sRAGE, esRAGE or HMGB1 in serum of patients with TETs, TH, MG and healthy volunteers. All ELISA tests were performed according to the manufacturers' instructions: sRAGE (Human RAGE Duoset, RnD Systems, Minneapolis, MN, USA), esRAGE (B-Bridge International Inc., CA, USA) and HMGB1 (IBL International GmbH, Hamburg, Germany). Researchers performing the assays and data analyses were blinded to the groups associated with each sample.

### Statistical methods

Statistical analysis of data was performed using SPSS software (version 20; IBM SPSS Inc., IL, USA). The concentrations of proteins in serum of patients with TETs were compared to those of healthy volunteers using Student's *t* test or One-way ANOVA for normal (Gaussian) distributions. Kruskal-Wallis rank test or Mann-Whitney U test was used to evaluate non-normal distributions. All data were reported as mean (median) ± standard deviation (and standard error mean). *Post hoc* comparisons were computed with the Tukey correction. Pearson's *χ^2^* test for independence was used for analysis of categorical data, such as sex differences. Spearman's rank correlation test was used to assess correlations, such as: molecular expression and histology. The level of statistical significance was set at <0.05 (two-tailed p-values).

## Results

### The RAGE axis in TETs, TH and regular thymus

#### Expression of RAGE in TETs

**Figure 1:**
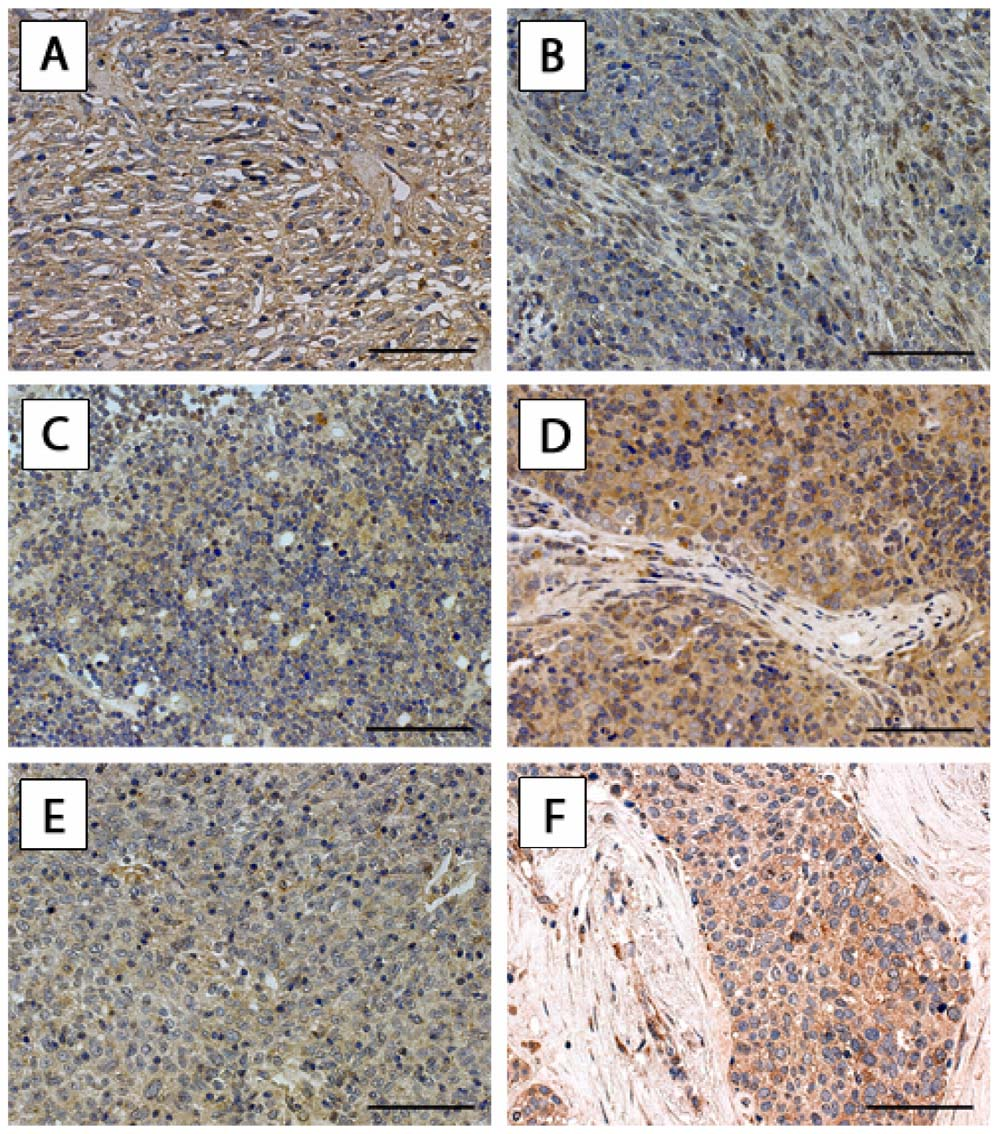
RAGE expression in TETs. Expression of RAGE in thymoma WHO type A (A), AB (B), B1 (C), B2 (D), B3 (E) and TC - SCC (F) is shown. Scale bar: 40 μm. *RAGE* receptor for advanced glycation endproducts, *TETs* thymic epithelial tumors, *WHO* World Health Organization, TC thymic carcinoma, *SCC* squamous cell carcinoma.

To test the hypothesis that RAGE is involved in thymic neoplasms, we employed immunohistochemical analysis for the detection of RAGE in tissue specimens of thymomas and thymic carcinomas. We found cytoplasmic staining for RAGE in all WHO types of thymomas (A, AB, B1, B2, B3) and thymic carcinomas (Fig. 1). Nuclear staining for RAGE was not detected. Semiquantitative analysis revealed strong expression of RAGE in thymic carcinomas and WHO type B2 thymomas. In comparison, the staining intensity in thymomas type A, AB, B1 and B3 was significantly lower (p<0.001; table 1). We did not find a difference in staining intensity in TETs of patients with or without MG (p = 1.000).

**Table 1:** Analysis of immunohistochemical staining of RAGE and HMGB1 for TETs.

TETs	Atypia	n(MG^+^)	RAGE Cytoplasmic		HMGB1 cytoplasmic		HMGB1 Nuclear	
WHO			%	Intens.* ^a^	%	Intens.* ^b^	%	Intens.* ^c^
**A**	None	6(3)	100.0	1.0[1-1]	0.0	-	100.0	3.0[3-3]
**AB**	None	3(0)	100.0	1.0[1-1]	0.0	-	100.0	2.7[2]-[3]
**B1**	Mild	6(3)	100.0	1.0[1-1]	33.3	3.0[3-3]	95.5	1.5[1]-[3]
**B2**	Clear-Cut	6(3)	100.0	2.5[2]-[3]	100.0	2.0[2-2]	100.0	1.5[1]-[2]
**B3**	Moderate^d^	6(3)	100.0	1.0[1-1]	75.5	1.0[1-1]	100.0	1.0[1-1]
**TC**	Clear-Cut	6(0)	100.0	3.0[3-3]	66.6	0.8[0-3]	33.3	0.8[0-3]

The percentage of epithelial tumor cells displaying staining for cytoplasmic or nuclear RAGE or HMGB1 is indicated. Cytoplasmic or nuclear staining intensity was scored as follows: score 0-3; 0, no staining; 1, weak; 2, moderate; 3, strong.

*TETs* thymic epithelial tumors, *RAGE* receptor for advanced glycation endproducts, *HMGB1* high mobility group box1, *WHO* World Health Organization, *Intens.* Staining intensity, *TC* thymic carcinoma;

^*^ mean[range]

a The staining intensity of cytoplasmic RAGE in TC and WHO type B2 thymomas was significantly higher than in thymomas of WHO types A, AB, B1 and B3 (p<0.001).

b Cytoplasmic staining intensity for HMGB1 was significantly higher in WHO type B thymomas and TC compared to WHO types A and AB (p<0.001).

c Nuclear staining intensity for HMGB1 was significantly higher in WHO type A and AB thymomas compared to the other WHO types (p<0.001).

d mild-to-moderate

#### Expression of the RAGE ligand HMGB1 in TETs

**Figure 2:**
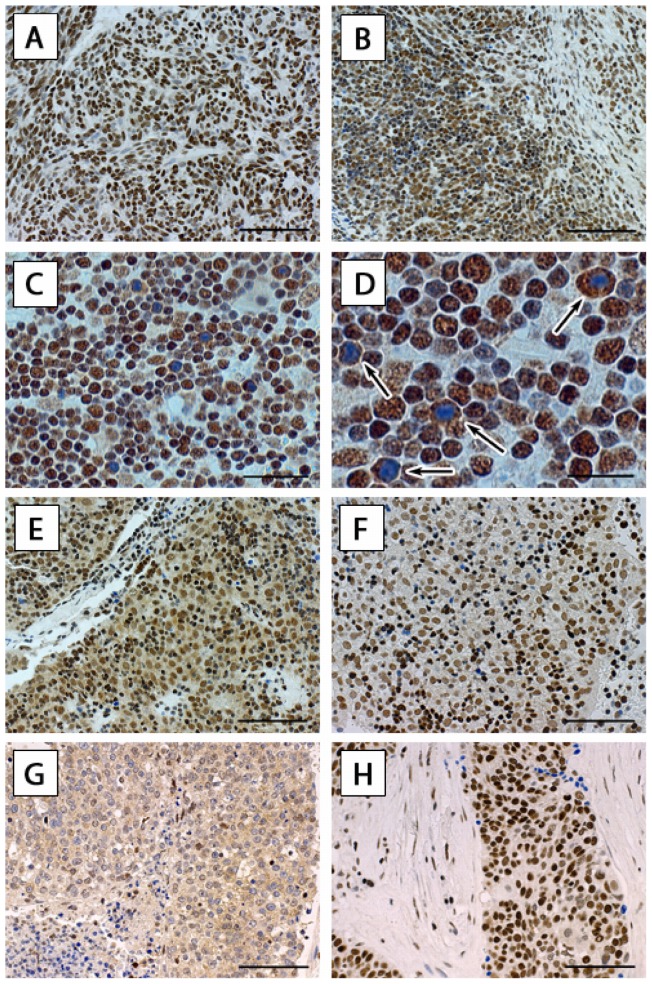
HMGB1 expression in TETs. Expression of HMGB1 in thymoma type A (A), B-component type AB (B), B1 (C) is shown. Scale bar: 40 μm. On this example of B1 thymoma lymphocytes are intermingled with few tumor cells. Focus on cytoplasmic staining: a larger magnification of a WHO type B1 thymoma is shown to better display “autophagic” tumor cells (tumor cells with brownish granular cytoplasmic and absent [only hematoxylin blue] nuclear staining). Scale bar: 20 μm (D). Analogously, a type B2 (E) and B3 (F) thymoma are shown. Two examples of TC (SCC (G) and (H)) are displayed – scale bar: 40 μm. *HMGB1* high mobility group box1, *TETs* thymic epithelial tumors, *TC* thymic carcinoma, *SCC* squamous cell carcinoma

Next we focused on the determination of the RAGE ligand HMGB1 in these tumors. Because of its role as a nuclear factor, staining for HMGB1 was expected in all cellular nuclei. We detected the strongest expression of HMGB1 in all nuclei of WHO type A and AB epithelial tumor cells. There was a gradual decrease in strength of HMGB1 nuclear staining from WHO type A (strong), type AB (moderate-to-strong), types B1 and B2 (weak-to-moderate), type B3 weak, culminating in the weakest expression in TC. Conversely, there was no expression of HMGB1 in the cytoplasm of WHO type A and AB thymomas, strong cytoplasmic staining in neoplastic epithelial cells of types B1, moderate in type B2, and weak in type B3 and TC (figure 2). The cytoplasmic staining intensity for HMGB1 was significantly higher in WHO type B1, B2, B3 thymomas and TC compared to WHO types A and AB (p<0.001), whereas the nuclear staining intensity for HMGB1 was significantly higher in WHO type A and AB thymomas compared to WHO types B1, B2, B3 and TC (p<0.001; table 1). While the majority of neoplastic epithelial cells of WHO type B1 thymomas displayed weak-to-moderate nuclear (95% of all nuclei stained positive for HMGB1) and strong cytoplasmic (33% of cells showed cytoplasmic staining) staining there were conspicuous epithelial cells in all B1 thymomas examined that showed clearly no nuclear staining for HMGB1 (figure 2). We did not detect a different staining intensity of HMGB1 in TETs of patients with or without MG (p = 1.000).

#### Expression of RAGE and HMGB1 in regular and hyperplastic thymus

**Figure 3:**
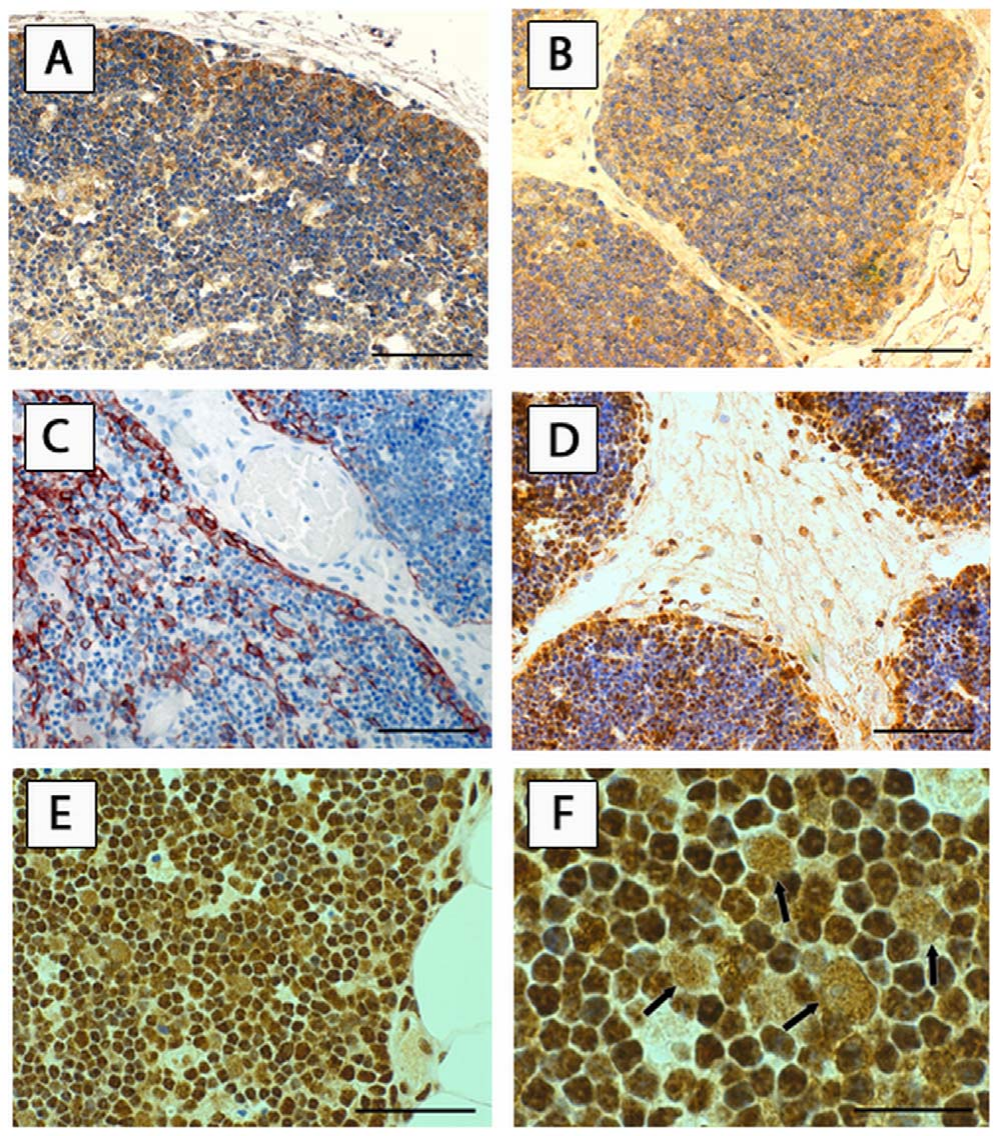
RAGE and HMGB1 expression in cTEC. Immunohistochemistry revealed strong expression of RAGE in subcapsular cTEC of fetal (A) and adult thymus (B). Scale bar: 40 μm. For comparison the staining pattern of cytokeratins 5 and 14 – markers of epithelial cell origin on fetal thymus is shown (C). The expression pattern for HMGB1 in cTEC of fetal (D, scale bar: 40 μm) and adult thymus (E, scale bar: 20 μm) is shown. (F) A larger magnification of E is shown. Scale bar 8 μm. Arrows in F indicate HMGB1 cytoplasmic staining in cTEC. *RAGE* receptor for advanced glycation endproducts, *HMGB1* high mobility group box1, *cTEC* cortical thymic epithelial cells

**Figure 4:**
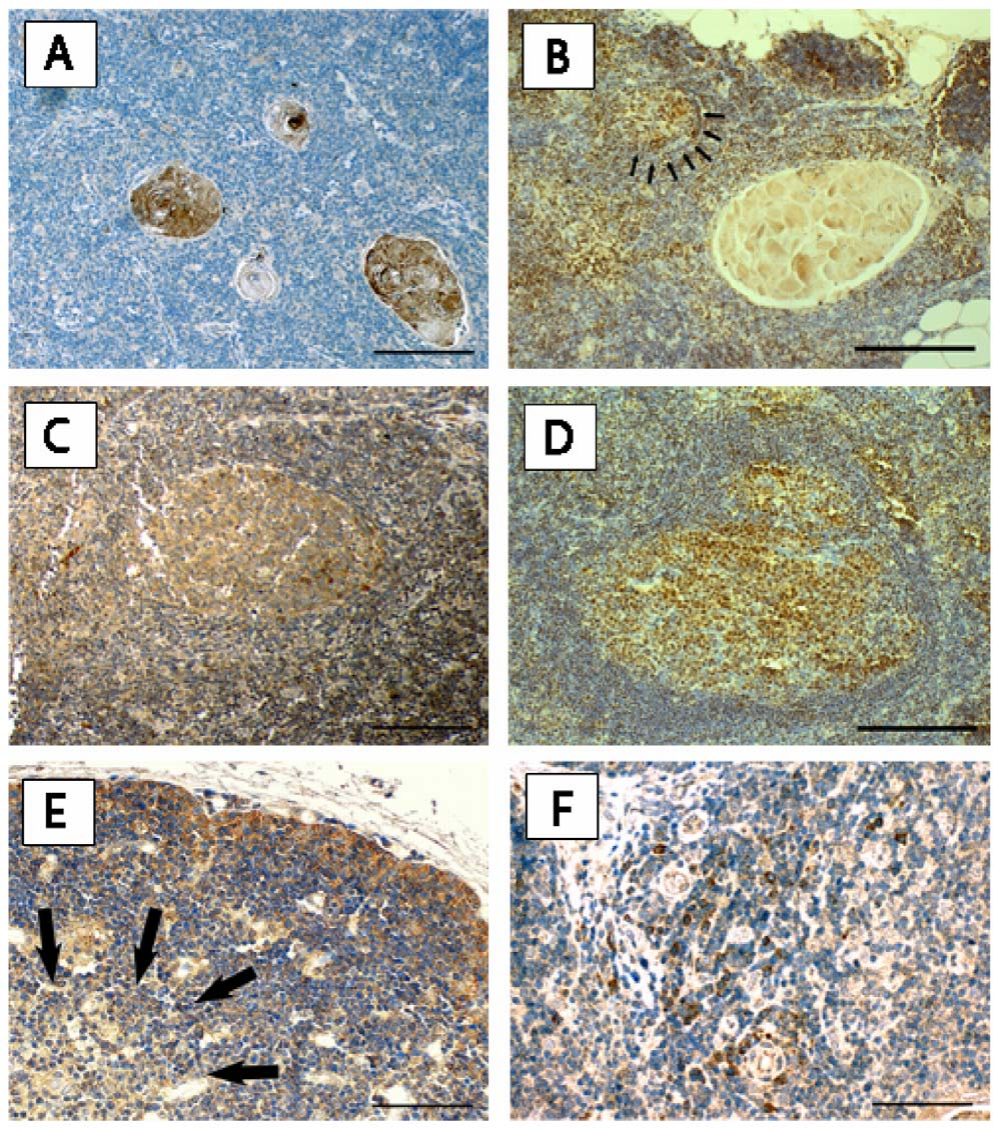
RAGE and HMGB1 expression in regular thymic morphology and MG. Hassall's corpuscles stained with antibodies to RAGE (A), and HMGB1 (B, small arrows point to small GC) are displayed. Scale bar: 80 μm. Similarly, RAGE (C), and HMGB1 (D) staining in GC of MG patients (scale bar: 80 μm); and RAGE expression in thymic medulla (E, arrows point to thymic medulla; scale bar: 200 μm) and macrophages (F, scale bar: 40 μm) of regular adult thymus are shown. *RAGE* receptor for advanced glycation endproducts, *HMGB1* high mobility group box1, *MG* Myasthenia gravis, *GC* germinal center.

We sought to delineate the (patho)-physiologic role of the RAGE axis in non-neoplastic thymic tissue. Nuclear staining for RAGE was not detected. In all thymuses of fetuses and children RAGE was expressed in subcapsular cortical epithelial cells (cTEC) of the outer thymic cortex (fig. 3). A marker of thymic epithelial cell origin (keratins 5 and 14, fig. 3C) was employed to identify subcapsular thymic epithelial cells. In the thymus of adults this staining pattern was found in 60.0% (9 out of 15) of specimens (table 2). The staining intensity seemed to correlate with age, as stronger RAGE expression was found in subcapsular cTEC of fetal thymus compared to infantile and adult thymus (table 2). Subcapsular RAGE expression was found in 50.0% of adult patients with (4 out of 8) and 74.4% without MG (5 out of 7), respectively (table 2, fig. 3). Analogously, HMGB1 expression was detected in cTEC of fetuses and adults with and without MG (fig. 3). Weak expression of both molecules was detected in thymocytes (fig. 3 A,B,D,E,F). Besides the described expression in cTEC we found RAGE and HMGB1 expressed in every single Hassall's corpuscle, thymic medulla and macrophages (fig. 4). In patients with MG and FTH, germinal centers markedly showed strong RAGE expression in plasma cells and weaker staining in centrocytes and centroblasts (fig. 4). Table 2 gives a semiquantitative summary of these results. Cytoplasmic HMGB1 was expressed in cTEC of fetuses and adults and germinal center cells of patients with MG (fig. 4). There was no difference in the type of the stained cells or their staining intensity in MG patients with and without immunosuppression.

**Table 2:** RAGE is expressed in different areas and cells of fetal, infant and adult thymus.

Group	histologic dx	case	age	sex	HC	medulla	GC	cTEC	MØ
**Fetal**	Regular thymus	1	12 w	f	+	+		+	++
	Regular thymus	2	22 w	f	+	+		++	+
	Regular thymus	3	20 w	f	+	+		++	+
	Regular thymus	4	22 w	m	+	+		++	+
**Infant**	Regular thymus	5	1 yr	f	++	+		+	+
	Regular thymus	6	13 yrs	f	+	+		+	+
**Adult**	Regular thymus	10	52 yrs	f	+	+		-	+
**No MG**	Regular thymus	11	23 yrs	f	+	+		+	+
	Regular thymus	12	29 yrs	m	+	+		+	+
	Regular thymus	13	53 yrs	f	+	+		+	+
	TTH	7	30 yrs	m	+	+		-	+
	TTH	8	53 yrs	f	+	+		+	+
	TTH	9	34 yrs	m	+	+		+	+
**Adult**	Regular thymus	14	32 yrs	f	+	+		+	+
**MG^+^**	Regular thymus	15	27 yrs	m	+	-		-	+
	Regular thymus	16	35 yrs	f	+	-		-	+
	FTH	17	36 yrs	m	+	+	+	+	+
	FTH	18	23 yrs	f	+	+	+	+	+
	FTH	19	27 yrs	m	+	+	+	+	+
	FTH	20	32 yrs	f	+	+	+	-	+
	FTH	21	40 yrs	f	+	+	+	-	+

The basic characteristics of each patient together with the tissue diagnosis (thymus regular for age, follicular thymic hyperplasia) and presence or absence of MG is detailed.

The intensity of staining for the different cells/structures was scored as follows (-, no staining; +, clearly positive; ++, very strong staining intensity).

*dx* diagnosis, *HC* Hassall's corpuscles, *medulla* thymic medulla, *GC* germinal center, *cTEC* subcapsular cortical thymic epithelial cells, *MØ* macrophages, *w* weeks of pregnancy, *yr(s)* year(s), *f* female, *m* male, *MG* myasthenia gravis, *regular thymus* thymic architecture regular for age, *TTH* true thymic hyperplasia, *FTH* follicular thymic hyperplasia

The moderate to strong cytoplasmic staining intensity of RAGE in B2 thymomas and thymic carcinomas compares to that found in cTEC, Hassall's corpuscles and macrophages of fetal and adult thymuses and centroblasts, centrocytes and plasma cells in GC of patients with non-thymomatous MG. Weak to moderate RAGE staining intensity of thymoma types A, AB, B1 and B3 compares to that of non-neoplastic thymocytes in AB, B1 and B2 thymomas and thymocytes in regular thymic tissue. We did not detect a difference of RAGE staining intensity in thymocytes of regular thymus or thymoma. The moderate cytoplasmic staining intensity of HMGB1 in B2 thymomas was comparable to that of cTEC, the weak intensity of B3 thymomas to that of thymocytes. The strong cytoplasmic staining intensity of B1 thymomas was not observed in non-neoplastic cells.”

### Concentration of sRAGE and its ligand HMGB1 in serum of patients with thymic abnormalities

Basic demographic data of patients with TETs, TH and volunteers whose serum analysis is described in the following paragraphs are detailed in table 3. There was no statistically significant difference in age (p = 0.110) and sex (p = 0.731) between patients with TETs and volunteers (table 3).

**Table 3:** Concentration of sRAGE, esRAGE and HMGB1 in serum of patients.

		TETs (n = 41)	Volunteers (n = 48)	*p* value
**Age in years**		53.4(55)±15.3(2.4)	47.6(46.5)±17.7(2.6)	*0.109* b
**F:M ratio n (%)**		19:22 (46.4:53.6)	24:24 (50:50)	*0.731* d
**Patients with Thymic Pathologies – categories:** ***sRAGE*** [pg/ml], ***esRAGE*** [pg/ml], ***HMGB1*** [ng/ml]			**One – way ANOVA** a **^,^** c **or** **independent samples T-Test** b	
**TETs**			**Volunteers** (**n = 45)**	***p*** ** value**
**sRAGE(n = 39)**			**sRAGE**	
246.8(206.7)±203.5(32.6)			364.8(318.9)±191.9(28.6)	*0.008* b
**esRAGE(n = 40)**			**esRAGE**	
357.4(328.2)±236.6(37.4)			412.9(421.4)±143.4(21.3)	*0.189* b
**HMGB1 (n = 39)**			**HMGB1**	
2.1(1.4)±2.0(0.3)			1.1(0.7)±1.3(0.2)	0.008 b
**TETs without MG**			**Volunteers** (**n = 45)**	
**sRAGE (n = 29)**				
224.5(196.3)±204.1(37.3)				*0.003* b
**esRAGE n = 29)**				
380.5(359.0)±260.2(47.5)				*0.490* b
**HMGB1(n = 28)**				
2.0(1.3)±2.1(0.4)				*0.025* b
**TETs**	**Thymic hyperplasia**		**Volunteers** (**n = 45)**	
**sRAGE(n = 39)**	**sRAGE(n = 28)**			
246.8(206.7)±203.5(32.6)	224.3(186.4)±153.8(29.1)			*0.003* c
**esRAGE(n = 40)**	**esRAGE(n = 28)**			
357.4(328.2)±236.6(37.4)	390.2(338.3)±188.4(35.6)			*0.414* c
**HMGB1 (n = 39)**	**HMGB1(n = 28)**			
2.1(1.4)±2.0(0.3)	1.6(1.0)±1.9(0.4)			*0.036* c
**Non invasive**	**Invasive**			
**sRAGE(n = 5)**	**sRAGE(n = 32)**			
468.5(410.0)±251.1(112.3)	208.8(167.5)±181.6(32.1)			*0.008* b
**esRAGE (n = 5)**	**esRAGE (n = 32)**			
375.1(400.9)±135.4(60.5)	350.2(319.9)±255.3(45.1)			*0.834* b
**HMGB1 (n = 5)**	**HMGB1(n = 31)**			
1.8(1.3)± 1.8(0.8)	2.1 (1.3)±2.1(0.4)			*0.795* b
**Thymoma**	**TC**		**Volunteers** (**n = 45)**	
**sRAGE (n = 22)**	**sRAGE(n = 17)**			
291.8(245.9)±221.0(47.1)	188.6(148.4)±166.9(40.5)			*0.008* c
**esRAGE (n = 23)**	**esRAGE (n = 17)**			
374.2(319.3)±287.7(60.0)	334.6(364.8)±147.2(35.7)			*0.346* c
**HMGB1 (n = 22)**	**HMGB1 (n = 17)**			
1.8(1.3)±1.8(0.4)	2.5(2.1)± 2.3(0.6)			*0.012* c
**Thymoma**			**Volunteers** (**n = 45)**	
**sRAGE (n = 22)**				
291.8(245.9)±221.0(47.1)				*0.267* b
**esRAGE (n = 23)**				
374.2(319.3)±287.7(60.0)				*0.559* b
**HMGB1 (n = 22)**				
1.8(1.3)±1.8(0.4)				*0.072* b
**TC**			**Volunteers** (**n = 45)**	
**sRAGE(n = 17)**				
188.6(148.4)±166.9(40.5)				*0.001* b
**esRAGE (n = 17)**				
334.6(364.8)±147.2(35.7)				*0.023* b
**HMGB1 (n = 17)**				
2.5(2.1)±2.3(0.6)				*0.009* b
**Thymoma**	**TC**		**Thymic hyperplasia**	
**sRAGE (n = 22)**	**sRAGE(n = 17)**		**sRAGE(n = 28)**	
291.8(245.9)±221.0(47.1)	188.6(148.4)±166.9(40.5)		224.3(186.4)±153.8(29.1)	*0.196* c
**esRAGE (n = 23)**	**esRAGE (n = 17)**		**esRAGE(n = 28)**	
374.2(319.3)±287.7(60.0)	334.6(364.8)±147.2(35.7)		390.2(338.3)±188.4(35.6)	*0.711* c
**HMGB1 (n = 22)**	**HMGB1 (n = 17)**		**HMGB1(n = 28)**	
1.8(1.3)±1.8(0.4)	2.5(2.1)±2.3(0.6)		1.6(1.0)±1.9(0.4)	*0.296* c

The basic characteristics of patients with TETs, TH and healthy volunteers are detailed. Characterization and categorization of patients with TETs according to type of TET, tumor invasiveness and presence of MG: the corresponding sRAGE, esRAGE and HMGB1 concentration in serum of patients are reported is mean (median) ± standard deviation (standard error mean).

a *Post hoc* analysis: please see the corresponding figures.

b independent-samples t-test

c one-way ANOVA

d Pearson's *χ^2^* test for independence

*f:m ratio* female:male ratio, *RAGE* receptor for advanced glycation endproducts, *sRAGE* soluble RAGE, *esRAGE* endogenous secretory RAGE, *HMGB1* high mobility group box1, *TETs* thymic epithelial tumors, *MG* Myasthenia gravis, *SEM* standard error mean, *n* number of patients, *TH* thymic hyperplasia, *TC* thymic carcinoma

#### Reduced sRAGE and increased HMGB1 serum levels in patients with TETs

**Figure 5:**
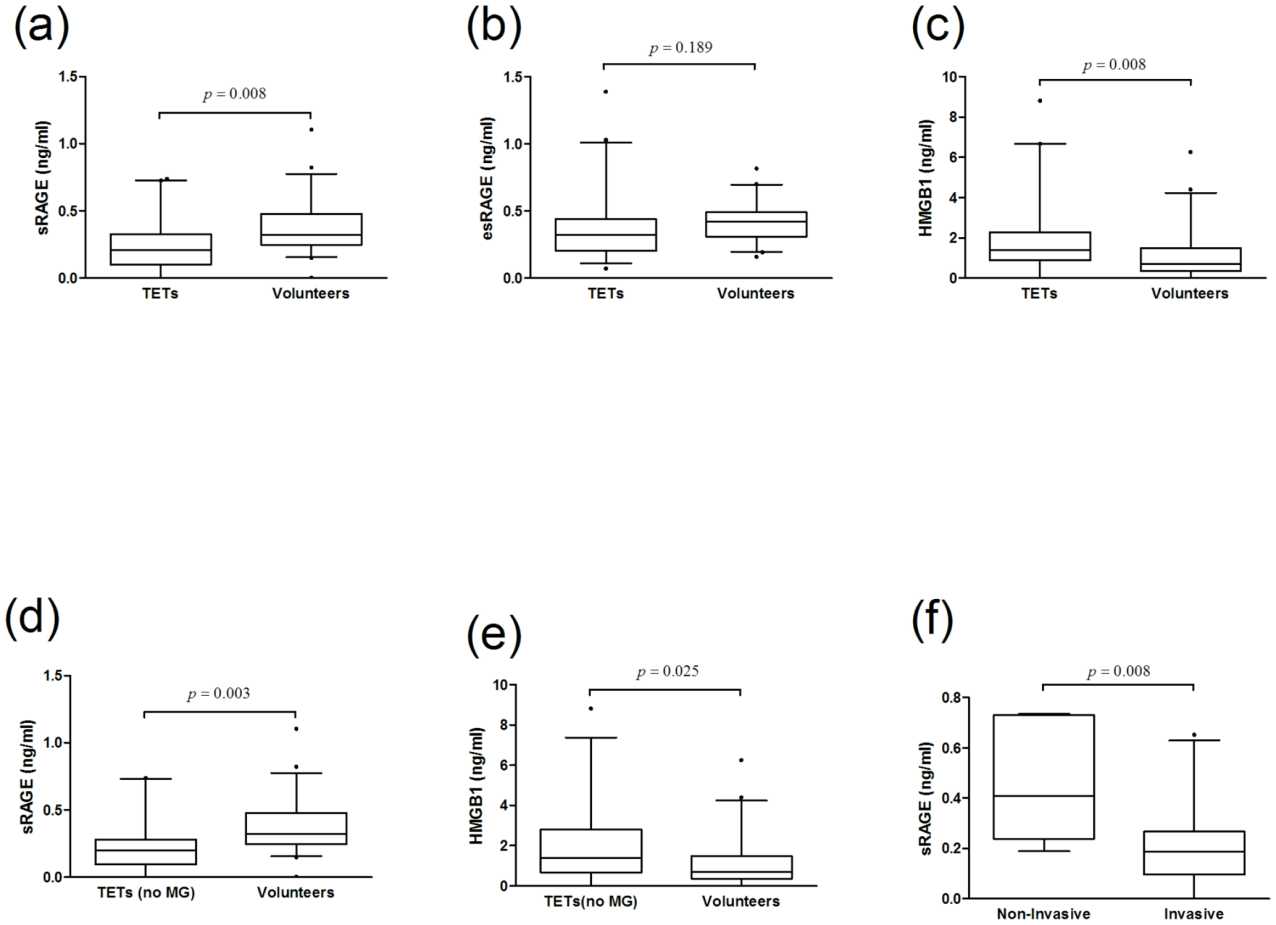
Serum concentrations of RAGE axis molecules in patients with TETs. Levels of sRAGE (a), esRAGE (b) and HMGB1 (c) in sera of patients with TETs including patients with paraneoplastic MG (MG n = 11) compared to healthy volunteers are shown. To rule out the influence of MG on levels of circulating sRAGE (d) and HMGB1 (e) in patients with TETs, patients with MG were excluded from this analysis. The levels of sRAGE in non-invasive (Masaoka-Koga stage I) and invasive TETs (Masaoka-Koga stages II-IV) are shown (f). *RAGE* receptor for advanced glycation endproducts, *sRAGE* soluble RAGE, *esRAGE* endogenous secretory RAGE, *HMGB1* high mobility group box1, *TETs* thymic epithelial tumors, *n* number, *MG* Myasthenia gravis.

We found significantly reduced serum concentrations of sRAGE in patients with TETs compared to healthy volunteers (sRAGE [pg/ml] 246.8±32.6 vs. 364.8±28.6; p = 0.008; fig. 5(a), table 3). The levels of splice variant esRAGE, however, were not different between patients with TETs and healthy controls (esRAGE [pg/ml] 357.4±37.4 vs. 412.9±21.3; p = 0.189; fig. 5(b), table 3). The concentration of HMGB1 in serum was significantly increased in patients with TETs (HMGB1 [ng/ml] 2.1±0.3 vs. 1.1±0.2; p = 0.008; fig. 5 (c), table 3). The above described results encompass the total population of patients with TETs in this study which includes 11 patients with MG. In the light of previously published reduced levels of sRAGE and esRAGE in patients with MG, we excluded patients with autoimmune disease (MG) from this analysis. In patients without MG the serum concentration of RAGE in TETs was significantly lower compared to volunteers (sRAGE [pg/ml] 224.5±37.3 vs. 364.8±28.6; p = 0.003) and the HMGB1 concentrations were significantly higher (HMGB1 [ng/ml] 2.0±0.4 vs. 1.1±0.2; p = 0.025). Again, esRAGE, showed no difference in serum expression between patients with TETs and volunteers (esRAGE [pg/ml] 380.5±47.5 vs. 412.9±21.3; p = 0.490; table 3 and figure 5). There was no statistically significant difference in age (p = 0.087), sex (p = 0.218) or serum concentrations of the indicated molecules when we compared MG^+^ (n = 10) with MG^−^ (n = 29) patients with TETs: sRAGE [pg/ml] 300.7±61.4 vs. 224.5±37.3, p = 0.359; esRAGE [pg/ml] 268.1±26.3 vs. 380.5±47.5, p = 0.190; HMGB1 [ng/ml] 1.9±0.5 vs. 2.0±0.4, p = 0.889.

#### RAGE serum concentration is lower in invasive TETs

We measured the sRAGE serum concentration in non-invasive (stage I) and invasive TETs (stage II, III and IV). SRAGE concentration was significantly higher in non-invasive than in invasive TETs (sRAGE [pg/ml] 468.5±112.3 vs. 208.8±32.1; p = 0.008; fig. 5).EsRAGE and HMGB1 serum concentrations, however, were similar in sera of non-invasive and invasive TETs (esRAGE [pg/ml] 375.1±60.5 vs. 350.2±45.1; p = 0.834; HMGB1 [ng/ml] 1.8±0.8 vs. 2.1±0.4; p = 0.795; table 3). We did not find a significant correlation between histologically classified WHO types of TETs (A, AB, B1, B2, B3 and TC) and sRAGE (correlation coefficient -0.213, p = 0.211) or HMGB1 (correlation coefficient 0.143, p = 0.413)

#### RAGE and HMGB1 serum concentration in patients with TH

**Figure 6:**
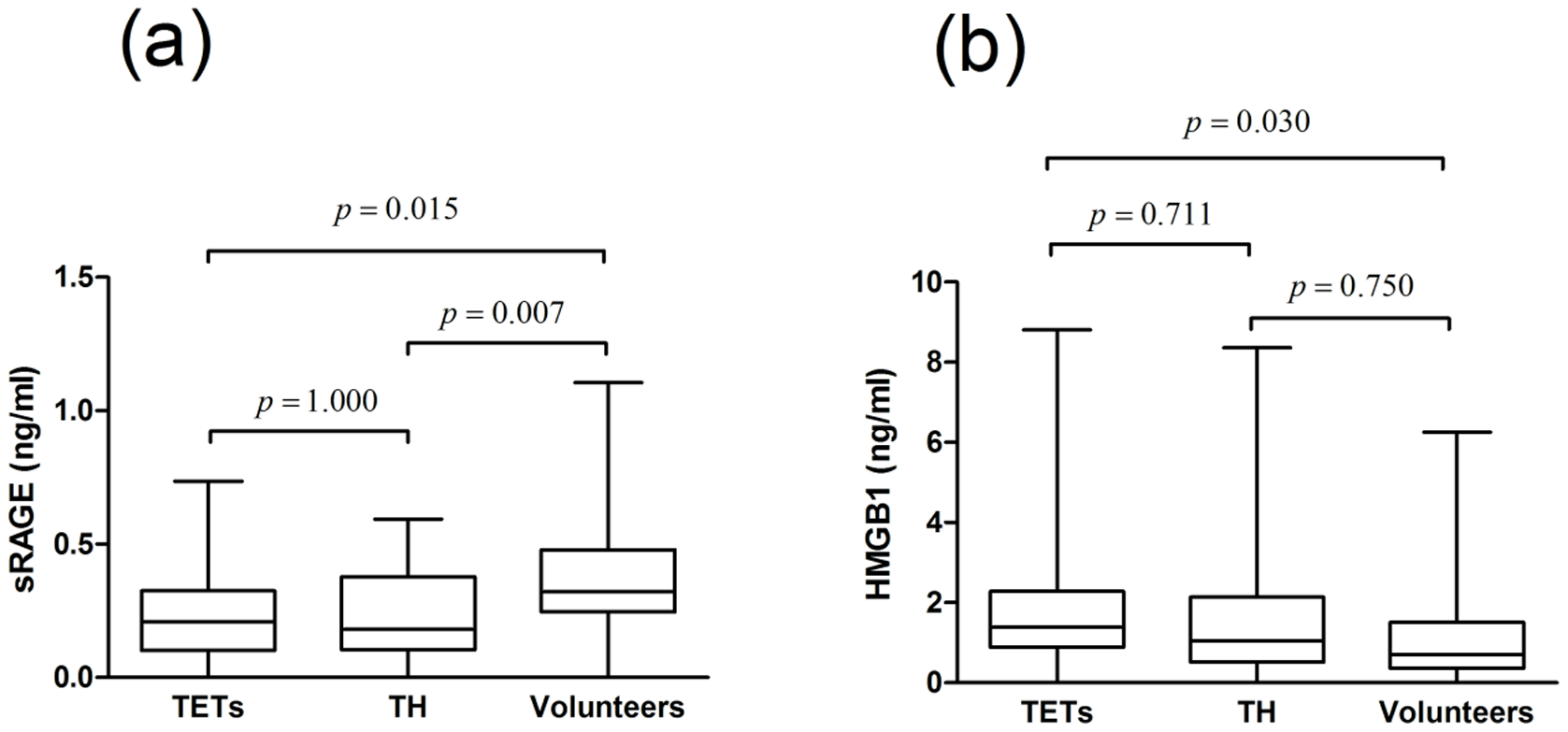
Serum concentrations of sRAGE and HMGB1 in benign and malignant thymic disease. The levels of circulating sRAGE (a), and HMGB1 (b) in serum of patients with TETs compared to patients with thymic hyperplasia and healthy volunteers are shown. *RAGE* receptor for advanced glycation endproducts, *sRAGE* soluble RAGE, *HMGB1* high mobility group box1, *TET* thymic epithelial tumor.

The levels of sRAGE in serum of patients with TETs and TH were significantly reduced compared to volunteers (sRAGE [pg/ml] TETs 246.8±32.6 vs. TH 224.3±29.1 vs. controls 364.8±28.6; p = 0.003). There was no difference between TETs and TH (p = 1.000; table 3, fig. 6 (a)). The serum expression of HMGB1 in patients with TETs was significantly higher compared to healthy volunteers (HMGB1 [ng/ml] TETs 2.1±0.3 vs. 1.6±0.4 vs. controls 1.1±0.2; p = 0.036). There was no statistically significant difference in HMGB1 levels between TETs and TH (TTH and FTH) or patients with TH and volunteers (table 3, fig. 6 (b)).

#### Emphasis on thymic carcinoma

**Figure 7:**
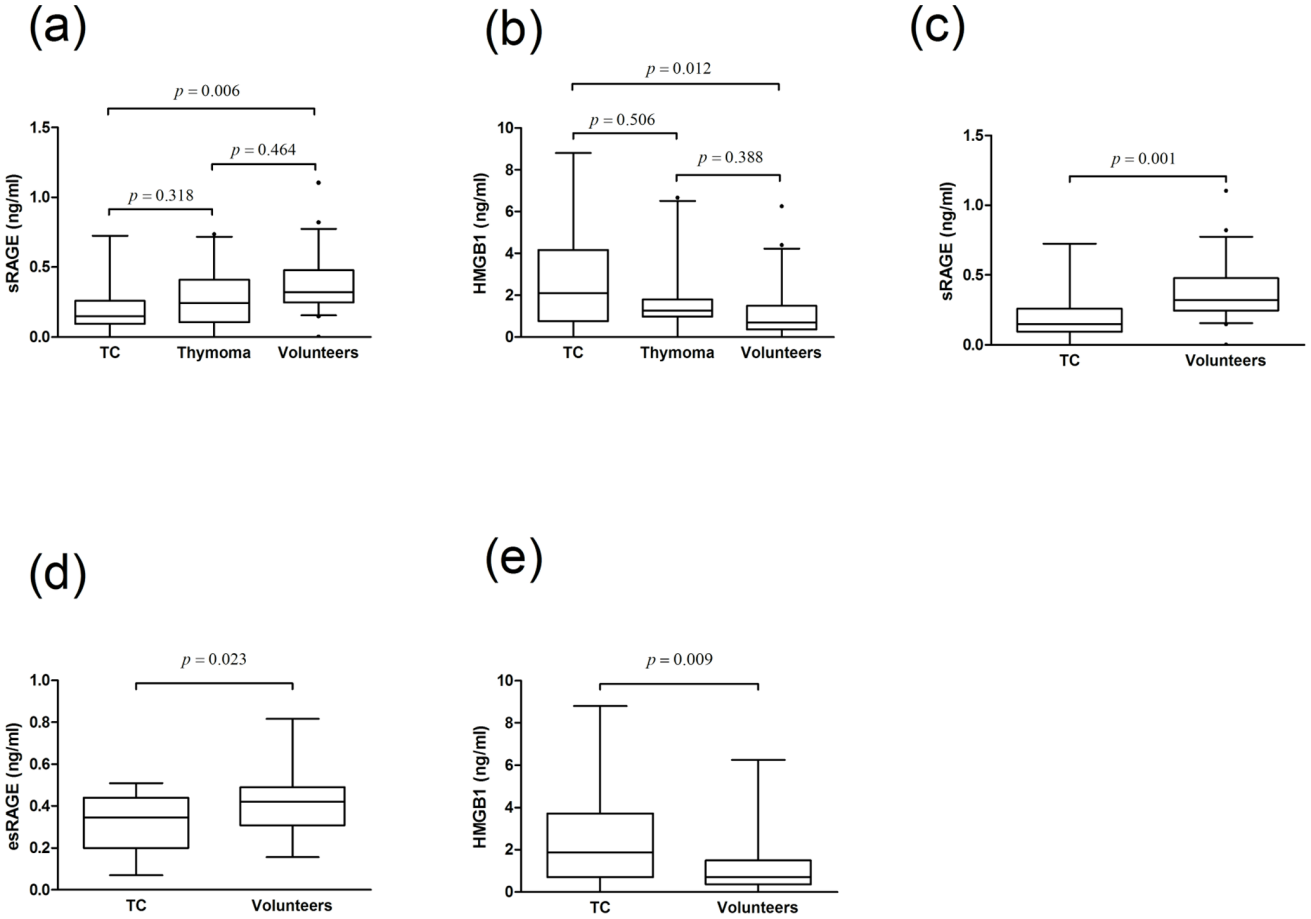
Serum concentrations of RAGE axis molecules in TC. Patients with TETs were separated into patients with thymomas and TC, and compared to healthy volunteers. Serum concentrations of sRAGE (a) and HMGB1 (b) are shown. Patients with TC were analyzed compared to volunteers for sRAGE (c), esRAGE (d), as well as HMGB1 (e). *TETs* thymic epithelial tumors, *RAGE* receptor for advanced glycation endproducts, *sRAGE* soluble RAGE, *esRAGE* endogenous secretory RAGE, *HMGB1* high mobility group box1, *TC* thymic carcinoma

Further stratification of patients with TETs into patients with TCs and thymomas compared to volunteers revealed significant differences in sRAGE and HMGB1 levels in serum but not in esRAGE levels (sRAGE [pg/ml] TC 188.6±40.5 vs. thymoma 291.8±47.1 vs. control 364.8±28.6; p = 0.008; fig. 7 (a); HMGB1 [ng/ml] TC 2.5±0.6 vs. thymoma 1.8±0.4 vs. control 1.1±0.2; p = 0.012; fig. 7 (b); table 3).

When we analyzed TCs and controls separately, we did not only find a highly significant reduction in sRAGE but also in esRAGE levels (sRAGE 188.6±40.5 vs. 364.8±28.6; p = 0.001; fig. 7(c); esRAGE 334.6±35.7 vs. 412.9±21.3; p = 0.023; fig. 7(d)). HMGB1 expression was again significantly elevated (HMGB1 2.5±0.6 vs. 1.1±0.2; p = 0.009; fig. 7(e)). The separate analysis of serum expression of these 3 molecules in patients with thymomas compared to volunteers did not reach significance (table 3).

The analysis of TC vs. thymoma vs. TH revealed no significant differences. The results for sRAGE were as follows: One-way ANOVA: p = 0.196; TC vs. thymoma: p = 0.250, thymoma vs. TH: 0.591, TC vs. TH: p = 1.000. The results for HMGB1 were as follows: One-way ANOVA: p = 0.296; TC vs. thymoma: p = 0.729, thymoma vs. TH: 1.000, TC vs. TH: p = 0.385.

## Discussion

Our findings are the first implication of a role for RAGE and its ligand HMGB1 in patients with TETs and non-neoplastic thymic disease, as well as in thymus of different developmental stages. This was evidenced by measurements in neoplastic and non-neoplastic thymic tissue and systemically in serum of patients.

RAGE and HMGB1 were differentially expressed in TETs. Thymoma types A and AB were shown to have a favourable prognosis compared to the other types [37]. WHO types A and AB showed weak staining intensity for RAGE, strong nuclear staining and no cytoplasmic staining for HMGB1. The observed progressive loss of HMGB1 in the nucleus of cancer cells from WHO type B thymomas and thymic carcinoma and conversely the increase of cytoplasmic staining intensity, starting with no expression of HMGB1 in the cytoplasm of epithelial tumor cells of WHO type A and AB thymomas, strong expression in B1 thymomas, moderate expression in type B2, and weak expression in type B3 and some thymic carcinomas; let us hypothesize that there could be a misbalance in the regulation of autophagy and apoptosis towards autophagy of malignant epithelial tumor cells. Autophagy is a process that ensures cell survival in the presence of DNA damage, starvation or other stressors. Apoptosis (programmed cell death) on the other hand eliminates damaged or mutated cells. Recently, it has been shown that HMGB1/p53 complexes regulate the balance between cell death and survival in human colorectal cancer cells lines (HCT116). The knockout of p53 in HCT116 cells increased the expression of cytosolic HMGB1 and induced autophagy [38].

The loss of nuclear and gain of cytoplasmic HMGB1 goes along with increasing atypia of different WHO typesof TETs [37]. Similarly, the highest RAGE expression was found in TETs with clear-cut atypia, namely WHO type B2 thymomas and thymic carcinomas. These findings are in accordance with a study on patients with colorectal adenomas where a significantly higher RAGE positivity was reported in colorectal adenomas with severe atypia [39].

Cytoplasmic coexpression of RAGE and HMGB1 in type B thymomas and TC – TETs with a worse prognosis compared to types A and AB – is not surprising when looking at what was found in malignancies of other origins. In patients with colorectal cancer the coexpression of RAGE and HMGB1 was associated with invasion and metastasis [40]. Higher coexpression of HMGB1 and RAGE in clear cell renal cell carcinoma positively correlated with tumor size, grade and clinical stage. HMGB1 mediated the progression of clear cell renal cell carcinoma via ERK1/2 activation, which was partially mediated by RAGE [30].

Lower systemic levels of sRAGE as observed in patients with TETs, particularly in TC, were reported in several malignant diseases. In a prospective case-cohort study on finish male smokers the concentration of sRAGE in serum of patients with pancreatic cancer was significantly lower than in smoking control subjects. The levels of the ligand CML-AGE were not associated with pancreatic cancer [29]. Another epidemiologic study found a lower risk of colorectal cancer in male smokers when prediagnostic serum sRAGE was upregulated [31]. Proinflammatory genes, including RAGE were over-expressed in central regions of human glioblastomas. This proinflammatory phenotype was suggested to be relevant for malignant progression [32]. RAGE expression was down-regulated in primary nasopharyngeal carcinomas, but up-regulated in metastases to cervical lymph nodes [33].

We found the strongest expression of RAGE in tissue of thymic carcinoma and WHO type B2 thymomas and weaker expression in other WHO types. In serum of patients the lowest levels of sRAGE were detected in patients with thymic carcinoma and higher levels in patients with thymomas. This inverse relationship between high cellular RAGE expression on the cell surface and low soluble receptor levels in serum may be directly interpreted as shortage of the decoy that could physiologically scavenge ligands and thus prevent further ligand-RAGE effects on the epithelial tumor or other RAGE expressing cells. Soluble RAGE in serum indicates benign and malignant thymic abnormalities, whereas HMGB1 is only elevated in malignant thymic disease. Information about both molecules could be of help for clinicians in the evaluation of thymic abnormalities (anterior mediastinal tumors) in patients.

Patients with TETs have an increased risk for secondary extrathymic malignancies [41]. Could the epidemiologic association between thymic and extrathymic malignancies be linked to chronic inflammation through the RAGE axis or direct RAGE effects on tumorigenesis? With the current available knowledge about the RAGE axis in patients with cancer one can only speculate about the cause and effect of reduced levels of soluble RAGE in patients with TC. On the one hand, one might suspect heightened serum concentrations of soluble RAGE if the suspected source is TC. On the other hand, a malfunctioning immune system (cancer immunosurveillance [42], [43]) could be the source of physiologically too little amounts of the decoy receptors sRAGE and esRAGE in patients with TC. Carcinogenesis could be promoted through binding of HMGB1 (elevated in TC patients), that is not scavenged by sRAGE/esRAGE, to the cell-surface bound RAGE receptor on nascent transformed cells (thymoma, TC). Obviously, this answer is speculative and other indirect mechanisms/phenomena are possible. Mature T cells released from the thymus contribute to cancer immunosurveillance. In heart- and lung transplant recipients, patients with compromised T cell immunity to avoid rejection of their grafts, a 7.1 fold increase in cancer risk was observed compared to the general population [44]. Conversely, a significantly lower risk of developing cancer was observed in people with high natural lymphocyte cytotoxicity [45]. Pharmacological blockade of RAGE in a murine model of allogeneic heart transplantation delayed graft rejection [21]. Transfer of RAGE-deficient TCR-transgenic mice recognizing OVA residues 323–339 T cells into ovalbumin-immunized hosts resulted in reduced proliferative responses that were further diminished in RAGE-deficient recipients. RAGE-deficient T cells showed impaired proliferative responses in vitro to nominal and alloantigens, in parallel with decreased production of IFN-γ and IL-2 [22]. Further studies are needed to see if the results with RAGE deficient T cells can be translated to tumor antigens.

Masaoka-Koga stage I thymomas show no transcapsular invasion and are thus designated non-invasive [3]. Patients whose stage I thymomas are completely resected by surgery have been reported to have excellent survival with surgery alone. Neither adjuvant radio- nor chemotherapy is recommended. Because of the possibility of late recurrence follow-up for more than 10 years is recommended [46]. The Masaoka-Koga staging system can only be applied after pathological evaluation of the resected specimen. Our results indicate that sRAGE could distinguish non-invasive from invasive TETs in the preoperative setting to aide decisions about the necessity of neoadjuvant therapy in cases of radiological suspicion of invasion.

To our knowledge RAGE and HMGB1 expression in normal human thymus and TH have not been described. The present findings show that RAGE and HMGB1 were expressed in subcapsular cTEC of normal thymic tissue and TH. In MG the proportion of patients missing subcapsular cTEC staining was larger (4 out of 8) than in patients without MG (2 out of 7). If this is of pathophysiological significance has to be determined in future studies. This staining pattern could be found in TTH without MG (2 out of 3), as well as in MG patients with FTH (3 out of 5). Evidence that cTEC mediate positive selection stems from experiments in mice whose MHC class II genes were deleted by targeted disruption [47]. The documented involvement of RAGE and/or HMGB1 in various autoimmune disorders, such as MG [9], systemic lupus erythematosus [48], [49], polymyositis [50], rheumatoid arthritis [51], [52] in conjunction with the new findings of these molecules in different cells in specific areas of thymus involved in positive (self MHC restriction) and/or negative selection (elimination of autoreactive T cells), namely thymic epithelial cells, raises the possibility of disturbances in central tolerance mechanisms linked to the RAGE axis.

RAGE was expressed in all Hassall's corpuscles. Located in the thymic medulla, Hassall's corpuscles are bodies of epithelial cells that have been assigned different roles in thymic physiology, such as phagocytic activity, roles in the maturation of medullary thymocytes or instruction of dendritic cells to induce CD4^+^CD25^+^ regulatory T cells in human thymus [53]. The expression of different cytokines in Hassall's corpuscles was described [54]. Germinal centers (GCs) could only be found in thymuses with FTH of patients with diagnosed MG. Centroblasts, centrocytes and plasma cells in GCs clearly expressed RAGE. GCs are not normally found in thymus, their place is in secondary lymphoid organs, as sites for mature B cells to proliferate and differentiate to plasma cells, for somatic hypermutation and class switching of antibodies during an immune response directed against infectious pathogens. Intrathymic (ectopic) GCs in MG patients contain B cells that undergo AChR-driven clonal expansion, somatic hypermutation and selection [55]. They further display features of normal GCs [56]. Further studies are warranted to investigate a possible role for RAGE in autoantibody production in intrathymic GCs of MG patients. Thymocytes showed a weaker expression for RAGE and HMGB1 than cTEC in all thymic tissues with some variance between individuals. During T-lymphocyte development the diverse repertoire of antigen receptors for T cells is created by the process of somatic recombination. A role for HMGB1 in V(D)J recombination has been described in conjunction with recombination activating genes (RAG) [57], [58].

While there were no age-related differences in the expression of RAGE in thymic medulla, Hassall's corpuscles, thymocytes or macrophages, the expression in cTEC in the subcapsular outer cortex detected in thymuses of fetuses was of much stronger staining intensity than in infants and adults. Moreover it was found in 75.0% (3 out of 4) of adult thymuses. Thymic involution is one of the most obvious observations paralleling declining immune function in the aging population. There is a decrease in naive and an increase in memory T cells with reduced function. These changes may be closely linked to steroid hormonal changes during puberty [59]. More supporting evidence from future studies is needed to rule out or confirm that RAGE is implicated in thymic involution.

Despite the improvements in the pharmacological treatment of patients with MG that have been made over the last decades, surgical thymectomy has evolved as empirical therapy and remains widely accepted as therapeutic modality providing partial or complete remission in selected patients with MG [10]. The molecular basis of this phenomenon is not completely understood. One possible explanation is intrathymic T cell sensitization and intrathymic production of antibodies to AChR epitopes [60]. The recent findings showing that sRAGE and esRAGE were reduced in patients with MG, in particular the observation that sRAGE was not influenced by pharmacological, including current immunosuppressive treatments for this autoimmune disease [9]; as well as the differentiated expression in cells and areas of thymus, in particular in GC cells in FTH in MG patients, let us hypothesize a role for the intrathymic cell surface bound receptor RAGE in the “intrathymic pathogenesis of MG” [60] and possibly a mechanism for the beneficial effects of thymectomy.

We have previously shown reduced levels of sRAGE and esRAGE in patients with MG [9]. Whereas these previous results stem from the analysis of MG patients without malignant neoplasms, the current study analyzed patients with TETs with or without MG. The lack of difference of sRAGE, esRAGE serum concentrations between MG^+^ and MG^−^ patients with TETs may be due to different pathophysiology of thymomatous and non-thymomatous MG [61], [62].Our decision to investigate the role of RAGE and one of its ligands, HMGB1, was fueled by the lack of information about these two molecules in human thymus and thymic disease in the peer-reviewed published literature. This is different for S100s. Antibodies to S100 are used routinely at our institution as diagnostic tool to identify dendritic cells in thymic specimens of human patients. During early ontogeny (4 months), S100^+^ thymic dendritic cell precursors populate the corticomedullary border and medullary region of thymus. Other thymic dendritic cell markers such as CD1a and Langerin appear later (at 5 months gestation) [63]. In thymus of newborns S100-protein^+^ cells, including interdigitating dendritic cells, were found in thymic medulla, at the corticomedullary junction, nearby and inside Hassall's corpuscles, but rarely in the cortex [64], [65]. The presence of S100^+^ antigen presenting cells in and around Hassal's corpuscles was also reported in adult (involuted) thymus [66]. S100^+^ (CD1a^+^, CD207^+^, CD11c^+^) positive dendritic cells were described in juvenile and immature Hassall's corpuscles of postnatal human thymus [67]. In a previous study S100β^+^ lymphocytes were found in 89% of thymomas. The degree of S100β^+^ lymphocyte infiltration correlated with thymoma stage [68].

In this study, sRAGE serum concentrations were lower than esRAGE concentrations in all experiments. One would expect higher sRAGE concentrations as the ELISA for sRAGE measures the pool of cleaved sRAGE and alternatively spliced esRAGE. This was also the case in our previous study on MG patients [9]. We are not suggesting that the absolute concentration values can be used to make judgments about the diagnosis of, for example TC. While comparative results (e.g. TC vs. volunteers) gained during one experiment could be repeated in separate ELISA experiments, the absolute values for the individual serum samples vary in our experienced hands with the recommended additional reagents from the manufacturer. So we never compared absolute values from samples measured with the sRAGE ELISA from different experiments. The intraassay coefficient of variation was 2.3%. The commercially available ELISA is sold for research use only and not for diagnostic purposes.

The described results about the RAGE axis in thymus raise new questions. Is there a role for other ligands of RAGE and other receptors of HMGB1? Several members of the S100/calgranulin family are involved in RAGE signaling. HMGB1 is also a ligand for TLR 4. TLR4 mRNA levels were found to be higher in thymic tissue of non-thymomatous MG patients and thymic tissue adjacent to thymomas compared to thymuses from cardiac surgery patients without MG [69]. The findings on RAGE, HMGB1 and their respective other ligands and receptors may warrant further study in autoimmune disease and cancer to elucidate therapeutic targets [70], [71].

One of the weaknesses of this study is the relatively small number of patients (especially for studies on patient serum) that does not allow further significant categorization of patients. Thymic neoplasms, as well as MG, are orphan diseases that need multidisciplinary and international efforts, such as current initiatives of the International Thymic Malignancies Interest Group (ITMIG) and Thymic Workgroup of the European Society of Thoracic Surgeons (ESTS). Cooperation with ITMIG and ESTS may soon allow answering some of the questions raised in large multicenter studies [72], [73].

In summary, we have shown the expression of RAGE and HMGB1 in distinct cells of regular thymus, germinal center cells of MG patients and TETs. Our immunohistochemical results were corroborated by alterations in the serum concentration of soluble RAGE variants and HMGB1. Because of the involvement of these molecules in autoimmune diseases, thymic and certain extrathymic cancers, these results may have substantial implications for diagnosis and/or treatment of not only thymic diseases. A more detailed understanding of the RAGE-HMGB1 axis and related molecules in thymic physiology and pathology is needed and warrants future study.
